# Association of health literacy and sleep problems with mental health of Chinese students in combined junior and senior high school

**DOI:** 10.1371/journal.pone.0217685

**Published:** 2019-06-07

**Authors:** Shi-chen Zhang, Rong Yang, Dan-lin Li, Yu-hui Wan, Fang-biao Tao, Jun Fang

**Affiliations:** 1 Department of Maternal and Child Health, School of Public Health, Anhui Medical University, Hefei, Anhui Province, P.R, China; 2 Anhui Provincial Key Laboratory of Population Health & Aristogenics, Hefei, Anhui Province, P.R, China; 3 Department of Toxicology, School of Public Health, Anhui Medical University, Hefei, Anhui Province, P.R, China; 4 Faculty of Pharmaceutical Science, Sojo University, Kumamoto, Japan; Medical University of Innsbruck, AUSTRIA

## Abstract

**Objectives:**

The aim of this study was to examine the association between health literacy (HL) and sleep problems with mental health of Chinese students in combined junior and senior high school.

**Methods:**

A cross-sectional study was conducted among seven hundred and seventy-five students from a combined junior and senior high school in Shenyang on December 16, 2016. HL, sleep problems, anxiety symptoms and depressive symptoms were measured by self-reported validated instruments. Multiple logistic regression models were used to examine the association of HL and sleep problems with mental health problems.

**Results:**

The prevalence of anxiety symptoms and depressive symptoms was 24.6% and 45.2%, respectively. Low HL was significantly associated with anxiety symptoms (*OR* = 2.457, *95%CI*: 1.493–4.045) and depressive symptoms (*OR* = 5.164, *95%CI*: 3.233–8.250). Sleep problems were significantly positively correlated with anxiety symptoms (*OR* = 4.237, *95%CI*: 2.831–6.341) and depressive symptoms (*OR* = 3.170, *95%CI*: 2.084–4.823). The students who had sleep problems with low HL had the highest risks of anxiety symptoms (*OR* = 11.440, *95%CI*: 5.564–23.520) and depressive symptoms (*OR* = 19.470, *95%CI*: 8.143–46.558).

**Conclusion:**

Our findings suggest that Chinese students in combined junior and senior high school who had sleep problems with low HL are at risk of exhibiting anxiety symptoms and depressive symptoms. Intervention programs of mental health problems should enhance HL level and improve sleep quality.

## Introduction

From a social-emotional developmental perspective, adolescence is a period of life that maybe specially vulnerable to physiological and psychological impact factors, for instance confusion and greater psychological pressure. It could even be the peak period for the onset of mental health problems [1–2]. Because emotion regulation mechanisms have not yet fully developed in adolescence, such as the ability to cope with negative emotions, adolescents are prone to many mental and behavioural problems [3]. Worldwide 10% to 20% of children and adolescents experience mental health problems, such as schizophrenia, depression, anxiety and intellectual disabilities, accounting for a large portion of the global burden of disease [4]. Anxiety and depression are the two most common psychological problems that can threaten adolescents’ mental health and academic performance [5]. Anxiety refers to the brain response to dangerous stimuli that an organism will actively attempt to avoid, which is not typically pathological as it is adaptive in many scenarios when it facilitates avoidance of danger [6]. Depression refers to the occasional sadness and gloom which accompanies the ups and downs of everyday life [7]. Childhood and adolescence are the core risk phase for the development of mental symptoms and syndromes, ranging from transient mild symptoms to full-blown mental disorders [6]. For example, depressive symptoms are known to escalate during adolescence, and adolescents who experience depressive disorder have an increased risk of mental illness in adulthood [8]. Thus, these conditions severely influence adolescents’ development, their educational achievement and their quality of life. However, these conditions could be contained efficiently or even reversed by improving level of mental health literacy (MHL) [9–10].

Sleep problems are related to a variety of mental health problems in both children and adolescents, impacting the ability to regulate emotions [11–12]. There is extensive evidence suggesting that adolescents having sleep problems report increased negative emotions [13]. Part of the relationship may be accounted for the effects of stress and emotional arousal interfering with sleep, although other evidence showing that sleep disruption can cause irritability and negative mood to adolescents [14–15]. Clinical epidemiological studies have also suggested that sleep disturbance is concomitant with mental disturbance [16–17]. Adolescents with disturbed sleep exhibit more signs of depression, anxiety, conduct problems, substance abuse, and suicidal behaviors [18–19]. Laboratory studies in particular have documented that impaired cognitive function, daytime sleepiness, and fatigue are consequences of sleep disturbance [20–21]. A longitudinal study revealed that sleep disturbance predicted increases in the prevalence of subsequent anxiety and depression [22]. In addition, the relationship between sleep and mental health problems in adolescents must be considered in both directions, which anxiety symptoms and depressed mood may be the most prevalent causes of sleep disturbance [23].

Mental health literacy is defined which “knowledge and beliefs about mental disorders, which aid their recognition, management or prevention” [24], it includes being knowledgeable about the preventive measures, clinical symptoms, treatment of mental disorders, self-support strategies and others experiencing from mental health status [10]. Growing evidences have shown that mental disorders can be halted and even reversed by elevating MHL level, particularly for those adolescents who exhibit depression and anxiety [25–26]. In addition, general health literacy (HL) which includes the domains of spiritual growth and stress management are closely related to MHL, which is also an important determinant of promoting mental health [27–28], and we thus used HL in the present study. Numerous studies observed that bad sleep quality (e.g. insufficient sleep, low night sleep duration, sleep delay) of children is associated with parental HL [29–31]. However, the health outcomes of adolescents are related to their own HL [32]. In addition, people with sleep disorders often exhibit lower HL, for instance limited HL was associated with obstructive sleep apnea [33–34]. Nevertheless, previous studies regarding the mental health problems focused on the effect of either HL or sleep quality independently, and the sizes of samples used in those studies were relatively small. Namely, no or very few studies described the interactive effect of HL and sleep disturbance on mental health problems. In this study, we aimed to examin the association between HL and sleep problems with mental health in Chinese students in combined junior and senior high school. In this regard, we hypothesized that low HL and sleep problems would be associated with the prevalence of mental health problems of students.

## Materials and methods

### Study participants

This study was approved by the Ethics Committee of Anhui Medical University. The study was performed in accordance with the Declaration of Helsinki. Permission for this study was requested from schools, parents, and students before completing the surveys. Participants received verbal and written descriptions of the study in detail while they provided their written informed consent. All selected subjects were informed of the purpose of the study and were assured confidentiality upon receipt of the questionnaire. This consent procedure was approved by ethics committees (approval number 20140087). Data were processed at a restricted location by using a personal unidentifiable code for each subject.

The sample population was selected by using convenience sampling in a combined junior and senior high school located in Shenyang, China on December 16, 2016. A total of 815 students (mean age of 15.58 ± 1.65 years, range from 11.67 to 20.61 years) were recruited in this study. Participants were from grade 7–12 in this school, resulting in the receipt of 775 (95.1%) valid questionnaires (questionnaires with missing data > 5% were discarded).

### Design of questionnaires

A self-administered questionnaire containing information on sociodemographic factors, HL, sleep problems and mental health was administered during a 20~30 min session in the classroom. The following socio-demographic characteristics were obtained: gender, grade, registered residence (rural or urban area), household structure, parents’ educational level, self-reported family economy and self-reported academic records and learning burden.

Sleep problems were measured by the Pittsburgh Sleep Quality Index (PSQI), which is a 19-item self-reported questionnaire that measures sleep quality and disturbances over a 1-month time interval [35]. This questionnaire encompasses seven domains including sleep quality, habitual sleep efficiency, sleep latency, sleep disturbances, medication use, sleep duration, and diurnal dysfunctions, over the past month. The sum of scores of these seven domains builds the PSQI global score, with higher scores indicating poor sleep quality. Recent studies demonstrated good reliability and validity of PSQI in children and adolescents as well. [36–38]. Based on the prior literature, PSQI global scores > 5 were defined as sleep problems in the present study [36]. The Cronbach’s α coefficient for the PSQI was 0.730 in this study.

The mental health of the participants was assessed, including anxiety symptoms and depression symptoms. The Self-Rating Anxiety Scale (SAS) and Self-Rating Depression Scale (SDS) were used to evaluate anxiety symptoms and depressive symptoms [39–40]. Both SAS and SDS survey scales contain 20 questions each. Answers are scored 1–4 points. The standard score was calculated by the total score multiplied by 1.25, which ranges from 25 to 100. Higher score indicated a higher level of mental disorder. The Chinese version of the SAS and SDS has been confirmed as a reliable and valid measure in the Chinese Population [36, 41]. Based on the Chinese norm for the SAS and SDS which reflects the subjective feelings of having anxiety or depressive symptoms, a total standard score of 50 and 53 was set as a cut-off point of anxiety symptoms and depressive symptom, respectively [42]. In this study, the Cronbach’s α coefficient for the SAS and SDS was 0.802 and 0.749, respectively.

The Chinese Adolescent Interactive Health Literacy Questionnaire (CAIHLQ) was used to assess HL, and the reliability and validity of CAIHLQ have been demonstrated in previous studies [43]. The CAIHLQ consists of 31 questions grouped into 6 domains as follows: (1) Physical activities (PA) of 6 items (e.g. ‘Following a planned exercise program.’); (2) Interpersonal relationship (IR) of 5 items (e.g. ‘Taking times with your family or friends.’); (3) Stress management (SM) of 6 items (e.g. ‘Balance time between study and play.’); (4) Self- actualization (SA) of 4 items (e.g. ‘Feeling each day is very meaningful.’); (5) Health awareness (HA) of 5 items (e.g. ‘Constricting sugars and food containing sugar.’); and (6) Dietary behavior (DB) of 5 items (e.g. ‘Eating 200–400 g of fresh fruit each day.’). Each item is rated on 5 selection categories (never and no desire, never but with desire, occasionally and irregularly, often, and routinely), and the total score is standardly converted to a score that ranges from 31 to 155, with lower scores indicating inadequate HL (see S1 File). In a previous study, the total questionnaire Cronbach’s α was 0.937, while the Cronbach’s α of each dimension was 0.752 to 0.898 [44]. In this study, the Cronbach’s α coefficient for the overall CAIHLQ was 0.919 and 0.736–0.865 for six subscales. According to the previous studies, students in this study were categorized as low, medium and high HL groups when their scores were < *P*_*25*,_ *P*_*25*_*—P*_*75*_ and > *P*_*75*_, respectively [43, 45–46].

### Statistical analysis

Statistical analysis was carried out by using SPSS ver. 23.0 for Windows (SPSS, Inc., Chicago, IL). The Chi-square analysis of variance was performed according to group differences, while Bonferroni-adjusted *P*-value were calculated. In this model, the *Cox&Snell R*^*2*^ coefficient and *Nagelkerke R*^*2*^ coefficient from different perspectives were calculated to evaluate the proportion of independent variables in the total variation of dependent variables. These two criteria were used to assess how well the model fit the data. Higher *Cox&Snell R*^*2*^ coefficient and *Nagelkerke R*^*2*^ value indicates a better predictive ability. We carried out univariate and multivariate logistic regression analyses to determine the abilities of the selected indicators. The multivariable regression models were used for major socio-demographic factors of household structure, self-reported family economy, academic record and learning burden. Statistical significance was set at *P* < 0.05.

## Results

### Univariate analysis

The overall CAIHLQ mean score for all participants was 106.49 ± 19.87, while the value of *P*_*25*_ and *P*_*75*_ were 93 and 120, respectively. Table 1 shows the prevalence of anxiety symptoms and depressive symptoms by frequency characteristics. One hundred and ninety-one (24.6%) and three hundred and fifty (45.2%) students reported anxiety symptoms and depressive symptoms. The total rate of depressive symptoms revealed statistically significant differences by family economy condition, self-reported academic record, learning burden, HL and sleep problems (*Bonferroni-adjusted P* < 0.05 for each). Statistically significant differences were also found in the total rate of anxiety symptoms by household structure, family economy condition, learning burden, HL and sleep problems (*Bonferroni-adjusted P* < 0.05 for each). No significant differences were found for the other socio-demographic variables (Table 1).

**Table 1:** Prevalence of anxiety symptoms and depressive symptoms among junior and high school students.

Variable	*n (%)*	Anxiety symptoms			Depressive symptoms		
		*n* (%)	*χ*^*2*^	*P value* ^*a*^	*n* (%)	*χ*^*2*^	*P value* ^*a*^
Gender			0.006	0.503		0.737	0.426
Male	412 (53.2)	102 (24.8)			192 (46.6)		
Female	363 (46.8)	89 (24.5)			158 (43.5)		
Grade			1.505	0.220		0.418	0.518
Junior school	252 (32.5)	69 (27.4)			118 (46.8)		
Senior high school	523 (67.5)	122 (23.3)			232 (44.4)		
Registered residence			0.017	0.897		0.001	0.987
Rural	188 (24.3)	47 (25.0)			85 (45.2)		
Urban	587 (75.7)	144 (24.5)			265 (45.1)		
Household structure			15.127	< 0.001		2.850	0.091
Only child	466 (60.1)	92 (19.7)			199 (42.7)		
More than one child	309 (39.9)	99 (32.0)			151 (48.9)		
Father’s educational level			0.018	0.892		1.621	0.203
< High school degree	439 (56.6)	109 (24.8)			207 (47.2)		
≥ High school degree	336 (43.4)	82 (24.4)			143 (42.6)		
Mother’s educational level			0.558	0.455		0.230	0.632
< High school degree	44 (56.8)	104 (23.6)			202 (45.9)		
≥ High school degree	335 (43.2)	87 (26.0)			148 (44.2)		
Self-reported family economy			6.681	0.105		10.205	0.018
Bad	63 (8.1)	24 (38.1)			38 (60.3)		
General	572 (73.8)	134 (23.4)			261 (45.6)		
Good	140 (18.1)	33 (23.6)			51 (36.4)		
Self-reported academic record			0.542	2.286		11.981	0.009
Bad	204 (26.3)	53 (26.0)			110 (53.9)		
General	412 (53.2)	102 (24.8)			183 (44.4)		
Good	159 (20.5)	36 (22.6)			57 (35.8)		
Self-reported learning burden			10.231	0.018		25.777	< 0.001
Light	65 (8.4)	16 (24.6)			21 (32.3)		
General	475 (60.9)	99 (21.0)			190 (40.3)		
Heavy	238 (30.7)	76 (31.9)			139 (58.4)		
HL			33.832	< 0.001		64.935	< 0.001
Low	184 (23.7)	75 (40.8)			125 (67.9)		
Medium	395 (51.0)	79 (20.0)			172 (43.5)		
High	196 (25.3)	37 (18.9)			53 (27.0)		
Sleep Problems			70.629	< 0.001		42.333	< 0.001
No	633 (81.7)	117 (18.5)			251 (39.7)		
Yes	142 (18.3)	74 (52.1)			99 (69.7)		
Total	775	191 (24.6)			350 (45.2)		

Note. Statistical methods: Chi-square test.

^*a*^ is Bonferroni-adjusted *P*-value.

HL: health literacy.

Table 2 shows the description of sleep quality according to PSQI. In terms of PSQI variables, the students with sleep problems had significantly higher prevalence of low HL, anxiety symptoms and depressive symptoms. All PSQI domains were significantly correlated with depressive symptoms. Namely, the students with bad subjective sleep quality, reduced sleep duration and efficiency, greater sleep disturbance, increased sleep latency and frequency of medicines had a higher prevalence of depressive symptoms (*Bonferroni-adjusted P* < 0.001for each). The students with good subjective sleep quality had significantly higher HL scores than students of other groups, while the same tendency was found on latency, disturbances, duration and diurnal dysfunctions (*Bonferroni-adjusted P* < 0.01for each). As expected, all PSQI variables were significantly correlated with anxiety symptoms except sleep efficiency, which is that the students with bad subjective sleep quality, reduced sleep duration, greater sleep disturbance, increased sleep latency and frequency of medicines had a higher prevalence of anxiety symptoms (*Bonferroni-adjusted P* < 0.001for each) (Table 2). In addition, the average length of sleeping was 7.55 ± 0.89 hr per night. There was no significant difference in sleep length between gender (*t* = 1.448, *P* = 0.148). The sleep length of students on weekdays was decreased with increasing grade (*F* = 40.956, *P* < 0.001).

**Table 2:** Description of students with sleep quality according to PSQI.

Variable	HL			Anxiety symptoms		Depressive symptoms	
	Low	Medium	High	No	Yes	No	Yes
Subjective sleep quality							
Very good	35 (16.4)	102 (47.7)	77 (36.0)	187 (87.4)	27 (12.6)	150 (70.1)	64 (29.9)
Good	98 (24.4)	221 (55.1)	82 (20.4)	319 (79.6)	82 (20.4)	221 (55.1)	180 (44.9)
Bad	38 (29.2)	62 (47.7)	30 (23.1)	70 (53.8)	60 (46.2)	48 (36.9)	82 (63.1)
Very bad	13 (43.3)	10 (33.3)	7 (23.3)	8 (26.7)	22 (73.3)	6 (20.0)	24 (80.0)
*χ*^*2*^	29.099			91.151		51.671	
*P value* ^*a*^	< 0.001			< 0.001		< 0.001	
Habitual sleep efficiency							
> 85%	144 (22.3)	326 (50.5)	176 (27.2)	501 (77.6)	145 (22.4)	376 (58.2)	270 (41.8)
75~84%	24 (29.6)	43 (53.1)	14 (17.3)	54 (66.7)	27 (33.3)	31 (38.3)	50 (61.7)
65~74%	7 (31.8)	14 (63.6)	1 (4.5)	14 (63.6)	8 (36.4)	9 (40.9)	13 (59.1)
< 65%	9 (34.6)	12 (46.2)	5 (19.2)	15 (57.7)	11 (42.3)	9 (34.6)	17 (65.4)
*χ*^*2*^	11.719			10.969		17.949	
*P value* ^*a*^	0.414			0.072		< 0.001	
Sleep latency							
≤15 minutes	40 (15.2)	139 (52.7)	85 (32.2)	226 (85.6)	38 (14.4)	170 (64.4)	94 (35.6)
16~30 minutes	94 (26.3)	185 (51.8)	78 (21.8)	269 (75.4)	88 (24.6)	200 (56.0)	157 (44.0)
31~60 minutes	36 (35.3)	45 (44.1)	21 (20.6)	60 (58.8)	42 (41.2)	37 (36.3)	65 (63.7)
≥60 minutes	14 (26.9)	26 (50.0)	12 (23.1)	29 (55.8)	23 (44.2)	18 (34.6)	34 (65.4)
*χ*^*2*^	23.959			40.689		32.716	
*P value* ^*a*^	0.006			< 0.001		< 0.001	
Sleep disturbances							
None	36 (15.8)	107 (46.9)	85 (37.3)	197 (86.4)	31 (13.6)	161 (70.6)	67 (29.4)
Mild	130 (26.7)	262 (53.8)	95 (19.5)	367 (75.4)	120 (24.6)	249 (51.1)	238 (48.9)
Moderate	16 (30.2)	23 (43.4)	14 (26.4)	18 (34.0)	35 (66.0)	14 (26.4)	39 (73.6)
Severe	2 (28.6)	3 (42.9)	2 (28.6)	2 (28.6)	5 (71.4)	1 (14.3)	6 (85.7)
*χ*^*2*^	30.497			72.133		47.554	
*P value* ^*a*^	< 0.001			< 0.001		< 0.001	
Medication use							
0	177 (23.8)	377 (50.6)	191 (25.6)	574 (77.0)	171 (23.0)	417 (56.0)	328 (44.0)
<1 time/week	4 (19.0)	14 (66.7)	3 (14.3)	9 (42.9)	12 (57.1)	8 (38.1)	13 (61.9)
1~2 time/week	2 (28.6)	4 (57.1)	1 (14.3)	1 (14.3)	6 (85.7)	0	7 (100)
≥3 time/week	1 (50.0)	0	1 (50.0)	0	2 (100)	0	2 (100)
*χ*^*2*^	4.810			33.263		13.693	
*P value* ^*a*^	1.704			< 0.001		0.009	
Sleep duration							
>7 hours	111 (20.0)	292 (52.7)	151 (27.3)	435 (78.5)	119 (21.5)	325 (58.7)	229 (41.3)
6~7 hours	56 (32.6)	76 (44.2)	40 (23.3)	122 (70.9)	50 (29.1)	85 (49.4)	87 (50.6)
5~6 hours	14 (32.6)	25 (58.1)	4 (9.3)	23 (53.5)	20 (46.5)	13 (30.2)	30 (69.8)
<5 hours	3 (50.0)	2 (33.3)	1 (16.7)	4 (66.7)	2 (33.3)	2 (33.3)	4 (66.7)
*χ*^*2*^	20.317			16.116		16.947	
*P value* ^*a*^	0.006			0.003		0.003	
Diurnal dysfunctions							
Very good	50 (17.7)	139 (49.1)	94 (33.2)	257 (90.8)	26 (9.2)	195 (68.9)	88 (31.1)
Good	38 (18.7)	115 (56.7)	50 (24.6)	155 (76.4)	48 (23.6)	124 (61.1)	79 (38.9)
Bad	46 (26.7)	100 (58.1)	26 (15.1)	114 (66.3)	58 (33.7)	78 (45.3)	94 (54.7)
Very bad	50 (42.7)	41 (35.0)	26 (22.2)	58 (49.6)	59 (50.4)	28 (23.9)	89 (76.1)
*χ*^*2*^	48.557			86.028		77.188	
*P value* ^*a*^	< 0.001			< 0.001		< 0.001	
PSQI global scores							
≤5 point	133 (21.0)	332 (52.4)	168 (26.5)	516 (81.5)	117 (18.5)	382 (60.3)	251 (39.7)
> 5 point	51 (35.9)	63 (44.4)	28 (19.7)	68 (47.9)	74 (52.1)	43 (30.3)	99 (69.7)
*χ*^*2*^	14.473			70.629		42.333	
*P value* ^*a*^	0.003			< 0.001		< 0.001	

Note. Statistical methods: Chi-square test.

^*a*^ is Bonferroni-adjusted *P*-value.

HL: health literacy; PQSI: Pittsburgh Sleep Quality Index.

### Multiple logistic regression analysis

**Fig 1:**
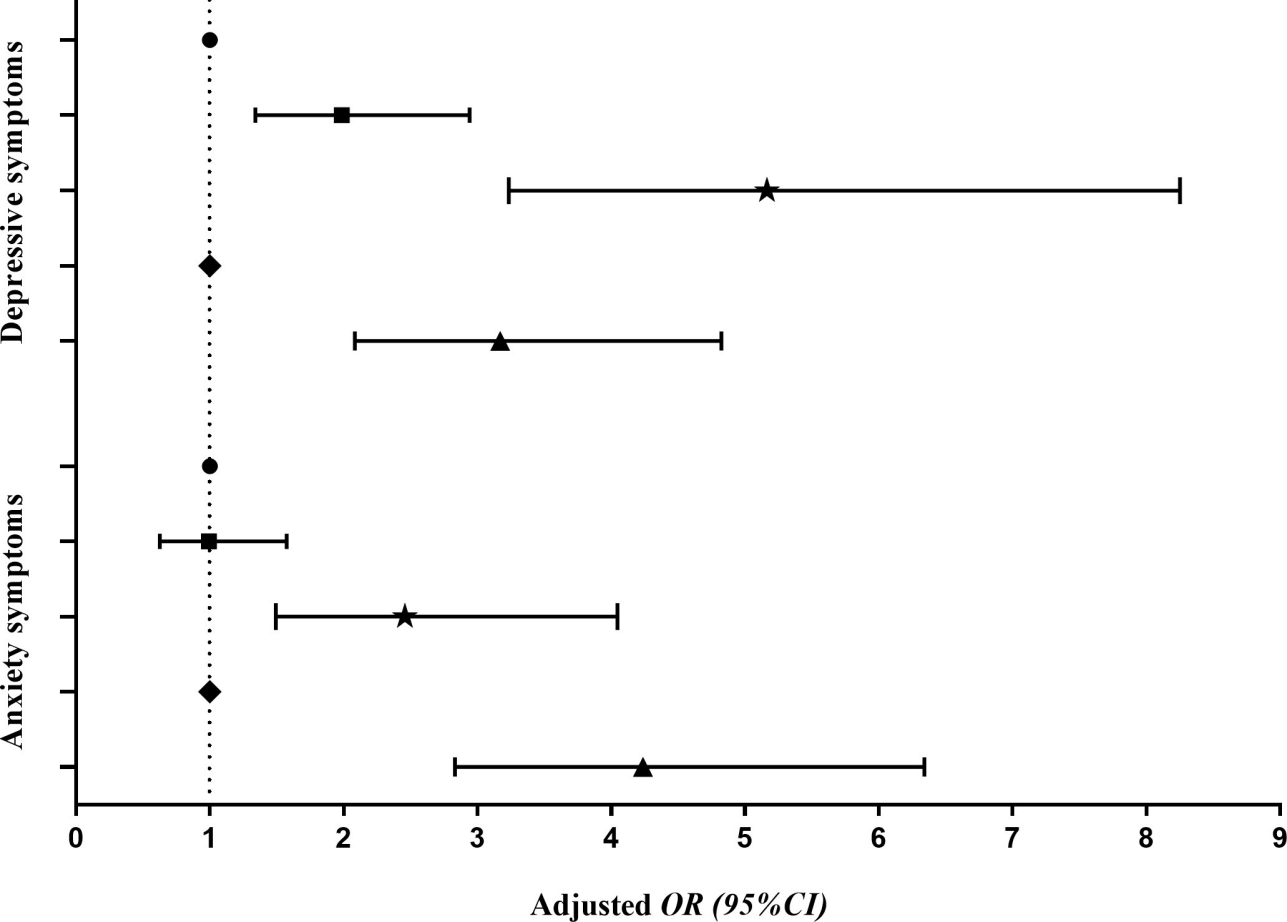
Associations of HL, sleep quality, anxiety and depressive symptoms among junior and high school students. Note. HL: health literacy. *OR*: odds ratio; *CI*: confidence interval. Adjusted for household structure, self-reported family economy, academic record and learning burden. ● High HL; ◼ Medium HL; ★ Low HL; ◆ No sleep problems; ▲ Have sleep problems].

Results from multiple logistic regression analysis indicated that, low HL was significantly associated with anxiety symptoms (*OR* = 2.457, *95%CI*: 1.493–4.045) and depressive symptoms (*OR* = 5.164, *95%CI*: 3.233–8.250), while sleep problems were positively correlated with anxiety symptoms (*OR* = 4.237, *95%CI*: 2.831–6.341) and depressive symptoms (*OR* = 3.170, *95%CI*: 2.084–4.823), after considering and adjusting household structure, self-reported family economy, academic record and learning burden (Fig 1).

**Fig 2:**
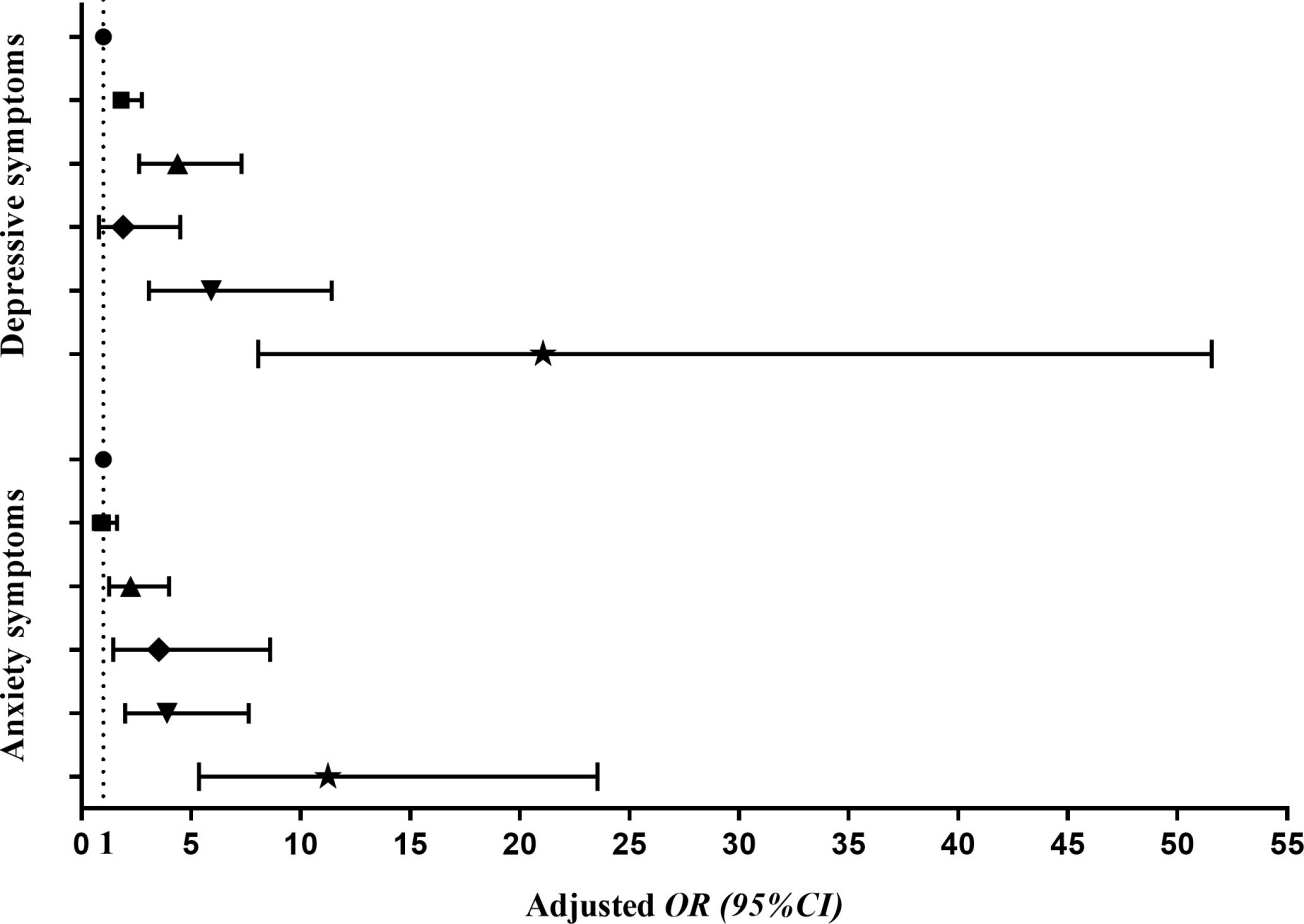
Odds ratio (95% *CI*) associated with the interaction of HL and sleep quality on anxiety and depressive symptoms among junior and high school students. Note. HL: health literacy. *OR*: odds ratio; *CI*: confidence interval. Adjusted for household structure, self-reported family economy, academic record and learning burden. ● No sleep problems+High HL; ◼ No sleep problems+Medium HL; ▲ No sleep problems+Low HL; ◆ Have sleep problems+High HL; ▼ Have sleep problems+Medium HL; ★ Have sleep problems+Low HL].

Multivariate logistic regression models were established with HL (high = 0, medium = 1, low = 2), sleep problems (no = 0, yes = 1), household structure (only child = 0, more than one child = 1), self-reported family economy (good = 0, general = 1, bad = 2), academic record (good = 0, general = 1, bad = 2) and learning burden (light = 0, general = 1, heavy = 2) as independent variables, while anxiety symptoms and depressive symptoms as dependent variables, respectively. The model fitted well, indicating significant high values of *Cox&Snell R*^*2*^ coefficient and *Nagelkerke R*^*2*^ coefficient that were 0.130 / 0.162 and 0.193 / 0.216 for anxiety symptoms and depressive symptoms, respectively. Fig 2 showed the interactions of HL and sleep quality with anxiety symptoms and depressive symptoms. The crude and adjusted *OR* (95% *CI*) were described for each group in comparison with the reference group (without sleep problems and high HL) for anxiety symptoms and depressive symptoms, respectively. The students with sleep problems and low HL had the highest risks of anxiety symptoms (*OR* = 11.440, 95% *CI*: 5.564–23.520, *P* < 0.001) and depressive symptoms (*OR* = 19.470, 95% *CI*: 8.143–46.558, *P* < 0.001). Same associations were also seen in the adjusted models (Fig 2).

## Discussion

In the present study, we found that HL and sleep problems are associated with mental health problems. Namely, low HL and sleep problems are correlated with the increased prevalence of anxiety symptoms and depressive symptoms.

Recent study has demonstrated that 14.58% of American adolescents (12–15 years) had self-reported sub-clinical internalizing problems, while 85.42% of them were clinically reported symptoms of depression, overall anxiety, or a specific anxiety disorder [based on the Screen for Child Anxiety and Related Disorders-Children (SCARED-C subscales), which include somatic/panic symptoms (e.g., ‘‘When I feel frightened, it is hard to breathe”), generalized anxiety (‘‘I worry about other people liking me”), separation anxiety (e.g., ‘‘I get scared if I sleep away from home”), social phobia (e.g., ‘‘I don’t like to be with people I don’t know well”), and school phobia (e.g., ‘‘I get headaches when I am at school”)[47]. In India, 25.5% of 7 904 students reported sadness and hopelessness that are considered as symptoms of depression, 8.6% reported persistent loneliness, and 7.8% reported insomnia that is related to anxiety [48]. In addition, the prevalence of all these markers of mental health problems significantly increased with age [48]. The prevalence of depressive symptoms among 19467 Chinese youths was 14.81% [49]. In the current study, a higher prevalence of 45.2% was observed, which may be due to different measurement tools. Considering that our participants were students of combined junior and senior high school, the results are reasonable. Many studies in China suggested that the students of combined junior and senior high school have more anxiety, depressive symptoms, interpersonal problems, character flaws, behavior and learning problems, etc., than normal middle school and high school students [50–51]. Our results indicated anxiety symptoms and depressive symptoms were associated with low family economy condition, and non-only child had more anxiety symptoms, which was similarly reported in previous studies [52–53]. Parents with bad economic conditions lack time and energy to communicate with their children, which may increase the occurrence and progression of children's mental health problems. Because of the one-child policy in China, the only children received much more caring and advantages, which is reflected as lower levels of psychological distress, compared to their peers with siblings [54].

Adolescents from East Asian countries rank of academic performance approximate to the top despite their relatively poor sleep habits [55]. As in China, students usually do not have adequate sleep because of academic pressure. Particularly, the total sleep duration for Chinese students decreasing to a severe extent when they enter high school with the increasing academic tasks. Participants in this study slept 7.55 h per night, a finding that is similar to those of previous studies in China [56]. Given that enough sleep is defined as 8–10 h each night regularly [57], the students of this study had a sub-optimal sleep duration. Adolescents with shorter sleep were more likely to report feeling diurnal dysfunctions, inattention, anxiety, and lower life satisfaction [58]. As reported, sleep disturbance coexists with psychotic symptoms, resulting in the positive correlation between sleep disturbance and anxiety and depressed moods [59]. Similarly, the findings of this study further strongly indicate the importance of quality sleep for adolescents’ mental health. Students' sleep quality could be greatly improved by educating students and their parents to elevate sleep related HL though a quiet sleep environment, no late-night social or other activities, low light exposure and increasing sleep time etc. [60]. To encourage better sleep practices in Chinese students, it may be better to focus on the importance of sleep for adolescents’ mental health.

Increasing evidences suggest that HL is associated with mental health in adolescents [25, 50, 61]. Our results indicated a strong interaction between inadequate HL and increasing anxiety symptoms and depressive symptoms. Moreover, low HL and sleep problems increase the risk of mental health problems independently and also synergistically (Figs 1 and 2). The results showed here strongly support the notion that continued efforts to enhance HL and to improve sleep quality are necessary for improving and maintaining mental health of junior and high school students. However, further investigations are needed.

Some limitations need to be acknowledge in the present study. Firstly, because of the cross-sectional design, causal relationships between correlates and outcomes could not be determined. Longitudinal studies are needed to explore the causal relationships. Secondly, this study did not directly use the evaluation tool of MHL, but the CAIHLQ included the domains of spiritual growth and stress management that are closely related to MHL, which can reflect the level of students' MHL to some extent. Finally, school-based sample did not focus on high-risk children or children who are not at school; meanwhile, due to the small sample size and self-report, selection and reporting biases could not be avoided.

## Conclusions

Although there are above limitations, the present study demonstrates the importance of associations of HL and sleep problems with mental health in Chinese junior and high school students. Our results suggest that low HL and sleep problems can cause anxiety symptoms and depressive symptoms gradually. Our findings may suggest that interventions are needed to enhance HL and sleep quality in junior and high school students. Further research is needed to measure the impacts of interventions to clarify the potential consequences on HL, sleep and mental health.

## Supporting information

S1 Appendix (Table A) Associations of HL, sleep quality, anxiety symptoms and depressive symptoms among junior and high school students. (Table B) Odds ratio (95% CI) associated with the interaction of HL and sleep quality on anxiety symptoms and depressive symptoms among junior and high school students.
(DOCX)

S1 File English version and Chinese version of CAIHLQ. (PDF)
