# Supplementary material for: Association of health literacy and sleep problems with mental health of Chinese students in combined junior and senior high school
Source: PLoS One. 2019 Jun 7;14(6):e0217685. doi: 10.1371/journal.pone.0217685 (PMC6555521; doi:10.1371/journal.pone.0217685)
Supplement: S1 Appendix — (Table A) Associations of HL, sleep quality, anxiety symptoms and depressive symptoms among junior and high school students. (Table B) Odds ratio (95% CI) associated with the interaction of HL and sleep quality on anxiety symptoms and depressive symptoms among junior and high school students. (DOCX) [file pone.0217685.s001.docx]

Appendix

Table A and Table B.

Table A Associations of HL, sleep quality, anxiety symptoms and depressive symptoms among junior and high school students

|  | Anxiety symptoms | |  | Depressive symptoms | |
| --- | --- | --- | --- | --- | --- |
|  | Crude *OR* (*95%CI)* | Adjusted *OR* (*95%CI)* |  | Crude *OR* (*95%CI)* | Adjusted *OR* (*95%CI)* |
| HL |  |  |  |  |  |
| High | 1.0 | 1.0 |  | 1.0 | 1.0 |
| Medium | 1.045 (0.666 - 1.639) | 0.992 (0.625 - 1.574) |  | 2.106 (1.439 - 3.084)^***^ | 1.985 (1.340 - 2.942)^**^ |
| Low | 2.579 (1.591 - 4.183)^***^ | 2.457 (1.493 - 4.045)^***^ |  | 5.327 (3.391 - 8.368)^*^^**^ | 5.164 (3.233 - 8.250)^**^ |
| Sleep problems |  |  |  |  |  |
| No | 1.0 | 1.0 |  | 1.0 | 1.0 |
| Yes | 4.409 (2.973 - 6.537)^***^ | 4.237 (2.831 - 6.341)^***^ |  | 3.212 (2.137 - 4.828)^***^ | 3.170 (2.084 - 4.823)^***^ |
| Household structure |  |  |  |  |  |
| Only child |  | 1.0 |  |  | 1.0 |
| More than one child |  | 1.894 (1.326 - 2.705) ^***^ |  |  | 1.188 (0.865 - 1.630) |
| Self-reported family economy |  |  |  |  |  |
| Good |  | 1.0 |  |  | 1.0 |
| General |  | 0.992 (0.609 - 1.615) |  |  | 1.206 (0.785 - 1.853) |
| Bad |  | 1.626 (0.796 - 3.321) |  |  | 1.550 (0.785 - 3.061) |
| Self-reported academic record |  |  |  |  |  |
| Good |  | 1.0 |  |  | 1.0 |
| General |  | 1.156 (0.675 - 1.981) |  |  | 1.615 (1.056 - 2.468)^*^ |
| Bad |  | 1.366 (0.845 - 2.206) |  |  | 2.138 (1.330 - 3.438)^**^ |
| Self-reported [learning](../../../../Program Files (x86)/Youdao/Dict4/7.5.0.0/resultui/dict/?keyword=learning)[burden](../../../../Program Files (x86)/Youdao/Dict4/7.5.0.0/resultui/dict/?keyword=burden) |  |  |  |  |  |
| Light |  | 1.0 |  |  | 1.0 |
| General |  | 0.936 (0.476 - 1.840) |  |  | 1.707 (0.915 - 3.187) |
| Heavy |  | 1.494 (0.745 - 2.996) |  |  | 3.348 (1.745 - 6.423)^***^ |

Note. HL: health literacy. *OR*: odds ratio; *CI*: confidence interval. Adjusted for household structure, self-reported family economy, academic record and [learning](../../../../Program Files (x86)/Youdao/Dict4/7.5.0.0/resultui/dict/?keyword=learning)[burden](../../../../Program Files (x86)/Youdao/Dict4/7.5.0.0/resultui/dict/?keyword=burden). ^*^ *P* < 0.05 ,^**^ *P* < 0.01 ,^***^ *P* < 0.001.

Table B Odds ratio (95% *CI*) associated with the interaction of HL and sleep quality on anxiety symptoms and depressive symptoms among junior and high school students

| Sleep problems | HL | Anxiety symptoms | | |  |  | Depressive symptoms | | |  |
| --- | --- | --- | --- | --- | --- | --- | --- | --- | --- | --- |
|  |  | *n* (%) | Crude *OR* (*95%CI)* | Adjusted *OR* (*95%CI)* | *P* value *^b^* |  | *n* (%) | Crude *OR* (*95%CI)* | Adjusted *OR* (*95%CI)* | *P* value *^b^* |
| No | High | 15 (10.2) | 1.0 | 1.0 | < 0.001 |  | 35 (22.4) | 1.0 | 1.0 | 0.007 |
|  | Medium | 30 (11.1) | 1.038 (0.618 - 1.745) | 0.959 (0.566 - 1.626) |  |  | 97 (35.9) | 1.968 (1.299 - 2.982)^**^ | 1.801 (1.176 - 2.759)^**^ |  |
|  | Low | 22 (24.7) | 2.549 (1.453 - 4.472)^**^ | 2.244 (1.261 - 3.993)^**^ |  |  | 47 (52.8) | 4.825 (2.941 - 7.917)^***^ | 4.380 (2.625 - 7.307)^***^ |  |
| Yes | High | 16 (40.0) | 4.290 (1.814 - 10.145)^**^ | 3.527 (1.446 - 8.601)^**^ |  |  | 18 (45.0) | 2.323 (1.016 - 5.313)^*^ | 1.900 (0.801 - 4.508) |  |
|  | Medium | 55 (44.0) | 4.576 (2.380 - 8.799)^***^ | 3.905 (2.000 - 7.624)^***^ |  |  | 75 (60.0) | 6.660 (3.523 - 12.588)^***^ | 5.926 (3.076 - 11.416)^***^ |  |
|  | Low | 53 (55.8) | 11.440 (5.564 - 23.520)^***^ | 11.242 (5.370 - 23.535)^***^ |  |  | 78 (82.1) | 19.470 (8.143 - 46.558)^***^ | 21.061 (8.602 - 51.565)^***^ |  |

Note. HL: health literacy. *OR*: odds ratio; *CI*: confidence interval. Adjusted for household structure, self-reported family economy, academic record and [learning](../../../../Program Files (x86)/Youdao/Dict4/7.5.0.0/resultui/dict/?keyword=learning)[burden](../../../../Program Files (x86)/Youdao/Dict4/7.5.0.0/resultui/dict/?keyword=burden).

*^b^ P*-value of interaction between HL and sleep quality on anxiety and depressive symptoms in multiplicative mode. ^*^ *P* < 0.05, ^**^ *P* < 0.01 , ^***^ *P* < 0.001 compared with referent.
