# Supplementary material for: Association of health literacy and sleep problems with mental health of Chinese students in combined junior and senior high school
Source: PLoS One. 2019 Jun 7;14(6):e0217685. doi: 10.1371/journal.pone.0217685 (PMC6555521; doi:10.1371/journal.pone.0217685)
Supplement: S1 File — (PDF) [file pone.0217685.s002.pdf]

Chinese Adolescent Interactive Health Literacy Questionnaire (CAIHLQ)

(Please select only one answer.)

Have you had done these things?	Never and no desire	Never but with desire	Occasionally and irregularly	Often	Routinely
1. Follow a planned exercise program.					
2. Exercise vigorous intensity for accumulate at least 60 minutes physical activity daily.					
3. Exercise moderate intensity for accumulate at least 60 minutes physical activity daily.					
4. Check own pulse rate after exercising immediately.					
5. Get exercise during usual daily activities (such as using stairs instead of elevators, by bicycle instead of by bus).					
6. Take part in leisure-time (recreational) physical activities (such as yoga, swimming, dancing).					
7. Get along well with your family.					
8. Maintain meaningful and fulfilling relationships with fiends or classmates.					
9. Show concern, love and warmth to others (such as friends, classmates, strangers).					
10. Get support from a network of caring people.					
11. Take times with your family or friends.					
12. Practice relaxation or meditation for 15-20 minutes daily.					
13. Get enough sleep.					
14. Concentrate on pleasant thoughts at bedtime.					
15. Use specific methods to control your stress (such as music,					

sports, and pour forth your feeling to someone).					
16. Balance time between study and play.					
17. Pace myself to prevent tiredness.					
18. Have courage to challenge new things.					
19. Work toward long-term goals in your life.					
20. Look forward to the future.					
21. Feel each day is very meaningful.					
22. Read or watch TV programs about improving health.					
23. Don't drink soft drinks.					
24. Read labels to identify nutrients, fats, and sodium content in packaged food.					
25. Limit use of sugars and food continuing sugar.					
26. Eat western fast food no more than twice a month.					
27. Eat 80-100 gram of meet each day (equal to the size of adult's palm 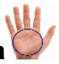)					
28. Eat 200-400 gram of fresh fruit each day (equal to the size of adult's 2-3 fist 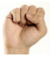).					
29. Eat 200-400 gram of vegetables each day (equal to the size of adult's 2-3 fist 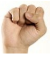).					
30. Eat 250-400 gram of cereal each day (equal to 3-4 small bowls of rice/noodle).					
31. Eat 2-3 servings of dairy products each day (equal to 250 ml of milk or 2 pieces of cheese).					

中国青少年互动性健康素养评定量表

单选题，每题请选择一个答案。

你做过以下这些事情吗？	没做过也没想做	没做过但想做	做过但没有规律	经常这样做	一直都这样做
**1.** 有计划的进行体育锻炼					
**2.** 每天会做 **60** 分钟（时间可累计）高等强度的运动（如打篮球、跳绳、踢足球、赛跑等）					
**3.** 每天会做 **60** 分钟（时间可累计）中等强度的运动（如中速跑、快走、乒乓球等）					
**4.** 运动后会立即自测脉搏					
**5.** 能够将运动融入于日常生活中（如走楼梯代替电梯、骑自行车代替坐车）					
**6.** 参加娱乐类的体育项目（如健美操、舞蹈、瑜伽等）					
**7.** 能与家人和睦相处					
**8.** 能与朋友或同学保持良好的人际关系					
**9.** 经常关心爱护、温暖他人（如同学、朋友、陌生人等）					
**10.** 当你遇到困难忧虑的事情时，能得到他人的帮助					
**11.** 会抽出时间与家人或朋友在一起					
**12.** 每天会抽出 **15～20** 分钟去放松心情、去散心或冥想					
**13.** 能保证充足的（自我感觉）睡眠					
**14.** 每天入睡前想些愉快的事情					
**15.** 平时会做一些消除压力的事情（如运动、倾诉、听音乐等）					
**16.** 能够平衡学习和业余娱乐的时间					
**17.** 生活中你会注意调节自己，防止疲劳					
**18.** 对新的事物勇于挑战					
**19.** 努力向着自己的人生目标前进					
**20.** 对自己的未来充满希望					
**21.** 每一天都感觉过得很有意义					

22. 经常看关于提高健康的宣传和电视节目					
23. 能做到不喝软饮料（如碳酸饮料、运动饮料、果汁饮料等）					
24. 买包装食品时，注意看包装袋后的营养成分表，确认脂肪、盐分等含量					
25. 控制摄取糖分过多的食物(如奶油蛋糕、膨化食品、饮料等)					
26. 每月吃西式快餐（如汉堡、薯条、炸鸡等）不超过 2 次					
27. 每天都吃 80～110 克的肉类（相当于 1 个普通成年人的手掌心的大小及厚度 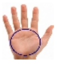）					
28. 每天都吃 200～400 克新鲜水果（相当 2～3 个普通成年人拳头大小 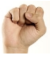）					
29. 每天都吃 200～400 克蔬菜（相当 2～3 个普通成年人拳头大小）					
30. 每天谷类(如米饭、面食等)都能吃 250～400 克（相当于 3～4 小碗米饭/面条或 3～4 个馒头）					
31. 每天都喝 2～3 份乳制品(如牛奶、酸奶、奶酪等)(相当于 250 毫升牛奶/酸奶或 2 片奶酪)					
